# Editorial: Regulation of PI3K/Akt signaling pathway: A feasible approach for natural neuroprotective agents to treat various neuron injury-related diseases

**DOI:** 10.3389/fphar.2023.1134989

**Published:** 2023-02-01

**Authors:** Yunhui Chen, Charles Hsu, Xiuping Chen, Heng Zhang, Wei Peng

**Affiliations:** ^1^ School of Basic Medicine/School of International Education/School of Pharmacy, Chengdu University of Traditional Chinese Medicine, Chengdu, China; ^2^ College of Medicine, Qatar University, Doha, Qatar; ^3^ Institute of Chinese Medical Sciences, University of Macau, Macau, China; ^4^ West China Hospital, Sichuan University, Chengdu, China

**Keywords:** PI3K/AKT, natural agents, neuron injury, neuroprotective effect, drug discovery

Neuron injury is closely associated with the development of various diseases, such as Alzheimer’s disease, spinal cord injury, ischemic stroke, and depression. The phosphoinositide-3-kinase (PI3K)/protein kinase B(Akt) signaling pathway is vital for neuron cell growth, development, and survival and can suppress cell apoptosis *via* downregulating proapoptosis proteins. In recent years, a growing body of research has suggested that natural agents are beneficial for preventing or treating neuron injury-related diseases *via* modulating the classical PI3K/Akt signaling pathway. This Research Topic “***Regulation of PI3K/Akt Signaling Pathway: A Feasible Approach for Natural Neuroprotective Agents to Treat Various Neuron Injury-Related Diseases***” provided an academic platform to discuss the novel natural neuroprotective agents and how they can be used to treat various neurodegenerative diseases regulating PI3K/Akt Signaling Pathway.

Using network pharmacology and *in vitro* experiments, Li et al. investigated the neuroprotective effects of Alpiniae oxyphyllae Fructus (AOF) on H2O2 stimulated PC12 cells, and the results suggested that AOF had the potential to treat Alzheimer’s disease by suppressing apoptosis induced by oxidative stress *via* the PI3K/Akt pathway. Liu et al. constructed an *in vitro* neuronal axotomy model to study the therapeutic effects of zinc oxide nanoparticles (ZnO NPs) on spinal cord injury (SCI) and found that ZnO NPs could act as a neuroprotective agent to reduce oxidative stress levels and rescue the neuronal apoptosis *via* the PI3K-Akt signaling pathway, providing new insights for SCI diagnosis and therapeutics. Yang et al. reported that Monomethyl lithospermate (MOL), a constituent of Shenxiong Tongmai granule, exerted a protective effect against neural damage caused by ischemic stroke in MCAO rats and OGR/R-induced SHSY-5Y cells, and its mechanism of actions attributed to the suppression of cell apoptosis through activating PI3K/Akt signaling pathway. Sun et al. adopted integrative approaches of network pharmacology and *in vivo* and *in vitro* experiments to study the antidepressant effect of essential oil from the roots of *Paeonia lactiflora* Pall (EOP) on corticosterone-induced depression and found that EOP might exert anti-apoptotic effects on hippocampal neurons through PI3K/Akt/Nrf2 signaling pathway. Tang et al. reported the antidepressant effects of the fruits of Zanthoxylum bungeanum Maxim. Essential oil (HEO) on chronic mild unpredictable stimulation (CUMS) mice *via* modulating the HPA axis and activating PI3K/Akt signaling pathway to reduce neuronal apoptosis. In addition, Xu et al. presented a review to describe and summarize recent research for sixteen natural terpenoids as neuroprotective agents associated with the PI3K/Akt pathway, providing a stepping stone for further research. And another review by Wang et al. illustrated that puerarin, a natural isoflavone extracted from the dried root of Pueraria montana var. Lobata (Willd.) Sanjappa and Predeep, could protect nerve cells and delay the progression of various neurological diseases through the PI3K/Akt signal pathway.

This Research Topic covers the latest scientific research on the regulation of PI3K/Akt signal pathway for natural products to treat different neuron injury-related diseases, including fundamental theory and experiments *in vitro* and *in vivo*. These investigations suggest that targeting PI3K/Akt signal pathway might be feasible for natural neuroprotective agents to manage such diseases as Alzheimer’s disease, spinal cord injury, ischemia stroke, and depression. However, other important neuro-injury related diseases have not been reported, such as Parkinson’s disease and epilepsy, and more investigations are warranted.

